# Delayed crises following benzodiazepine withdrawal: deficient adaptive mechanisms or simple pharmacokinetics? Detoxification assisted by serum-benzodiazepine elimination tracking

**DOI:** 10.1007/s00228-021-03205-x

**Published:** 2021-09-13

**Authors:** Anna Basińska-Szafrańska

**Affiliations:** Institute of Psychiatry and Neurology, Sobieskiego 9, 02-957, Warsaw, Poland

**Keywords:** Benzodiazepines, Detoxification, Elimination time, Delayed withdrawal symptoms, Serum-BZD, Addiction relapse

## Abstract

**Objective:**

Rapid relapses after successful withdrawal occur even in apparently motivated benzodiazepine (BZD)-dependent patients. Regardless of known personality or biological (re-adaptation) issues, the aim of this open-label, single-arm, seminaturalistic study was to search for any detoxification errors contributing to failures.

**Methods:**

The data came from 350 inpatients. Based on serum-BZD evolution criteria, the procedure was divided into four stages: substitution, accumulation, elimination and post-elimination observation. After switching the patients to a long-acting substitute (diazepam), to prevent data falsification due to unwanted overaccumulation, the doses were expeditiously reduced under laboratory feedback until accumulation stopped. With the start of effective elimination, the tapering rate slowed and was individually adjusted to the patient’s current clinical state. The tracking of both serum-BZD concentration and the corresponding intensity of withdrawal symptoms was continued throughout the entire elimination phase, also following successful drug withdrawal. Detoxification was concluded only after the patient's post-elimination stabilization.

**Results:**

Regardless of various initial serum-BZD concentration levels and the customized dose-reduction rate, and despite the novel lab-driven actions preventing initial overaccumulation, elimination was systematically proven to be protracted and varied within the 2- to 95-day range after the final dose. Within this period, withdrawal syndrome culminated several times, with varying combinations of symptoms. The last crisis occurrence (typically 2–3 weeks after withdrawal) correlated with the final serum-BZD elimination. The factors that prolonged elimination and delayed the final crisis were patient age, duration of addiction, adjunct valproate medication and elimination stage start parameters growing with former overaccumulation.

**Conclusions:**

The low-concentration detoxification stage is critical for patients’ confrontations with recurring withdrawal symptoms. Underestimated elimination time following drug withdrawal and premature conclusions of detoxification expose patients to unassisted withdrawal crises. Concentration tracking defines proper limits for medical assistance, preventing early relapses.

**Supplementary information:**

The online version contains supplementary material available at 10.1007/s00228-021-03205-x.

## Introduction

Despite the growing problem of benzodiazepine (BZD) addiction, the treatment principles remain unstructured, and as argued [1, 2], most studies on detoxification procedures do not meet the criteria for clinical trials. This results in a multitude of reviews, confirming the eclectic state of the art [3–5]. In turn, data are scarce regarding actual treatment efficacy and frequency of dropouts or early relapses.

Considering treatment failures, there are sustained concerns about delayed or protracted withdrawal symptoms [6–8], which may precipitate relapse. Up to now, these were attributed to individual inertia of adaptive mechanisms. The drug-induced adaptive changes that develop over years of regular use, with decreasing drug concentrations remain unbalanced until re-adaptation occurs [9–11]. An individually different rate of re-adaptation progress results in differentiated tolerance to detoxification.

Both clinical observations and theoretical considerations on symptoms following drug discontinuation (not only drugs of abuse) point to a low-concentration trigger [11–13]. Tapering is quite easy at high doses but is much more difficult in the low-dose phase [14]. In numerous detoxification (detox) schedules, gradation or/and elongation of tapering stages increases with diminished doses [11, 15]. Flexible approaches seem even more reasonable [16–19], as manipulating the rate of gradual dose reduction at each worsening of withdrawal symptoms seemingly adjusts the procedure to the individual’s re-adaptation course. Consequently, a patient’s good condition after drug withdrawal is taken as proof of complete post-detox re-adaptation, seemingly justifying the conclusion of a detox. In clinical practice, however, unexpected post-detox crises occur.

Conviction about the sometimes-protracted re-adaptation process leads to pressure to greatly prolong the drug-tapering procedure for months to years, especially when the treatment is carried out in ambulatory settings [15]. This prolongation, in turn, extends addiction-related harm [20].

However, it has been recorded in the past, although not commented on [12, 13, 21–23], that when detoxification is typically [24] preceded by replacing abused drugs with a slowly eliminating substitute BZD, then the crucial low-concentration phase may be involuntarily shifted far beyond drug withdrawal. Hence, it might be expected [25] that a delayed low-concentration phase would result in delayed withdrawal syndrome after the cessation of medical assistance. In such conditions, relapse becomes highly likely.

Hence, a distinction must be made between protracted symptoms due to an excessively slow re-adaptation process and delayed withdrawal symptoms associated with predictable pharmacokinetic (PK) issues. The latter, if recognized, might be avoidable by adjusting the procedure to elimination data. However, such a distinction has never been attempted in practice. Despite the knowledge about extensive individual variations in long-acting BZD elimination times, concentration tracking, unless for toxicological reasons, is currently observed rarely and only on a case-by-case basis [26, 27].

The data presented here come from a seminaturalistic study settled in conditions of a typical detoxification ward. What was novel was the routinely conducted serum-BZD check to track the real course of the detoxification process. The aim of this single-arm, open-label study was to investigate the relationship between BZD concentration evolution and detoxification crises, to clarify 1) whether PK phenomena predict the timing of crises 2) which factors are relevant and 3) do the results translate to clinical practice.

## Material and methods

### Patients

A total of 350 adult inpatients meeting the ICD-10 criteria for BZD dependence [29] were enrolled to the study in order of admissions. All the patients declared motivation to attain abstinence and were referred to the detoxification ward following one or more unsuccessful attempts in outpatient or inpatient setting.

In this ethically approved *(cf. Ethical statement)*, seminaturalistic study, the recruitment of patients, with their informed consent, was not targeted at those with no coexisting somatic and psychiatric conditions. In contrast, as is typical for CNS depressant addiction, the majority of patients reported a history of insomnia, anxiety or mood disorders as primary (pre-addiction) conditions. However, a stable clinical state in this regard was required. Consequently, the patients maintained their basic medications, but drugs significantly influencing BZD metabolism were noted. Many patients were also alcohol-dependent, but a state of stable abstinence (negative alcohol test result, no withdrawal symptoms) was required, without pharmacological support. Urine drug screening test was performed, as poly-drug users were excluded from this study.

### Procedure

Following popular approaches, the procedure involved a pre-detox replacement of the formerly used BZDs by a standard long-acting BZD (diazepam). As an innovation, actions were undertaken to prevent the unneeded accumulation of the substitute, which could influence both the patient’s state and the drug elimination course investigated in the study. To respect individualized metabolism and re-adaptation rates, the tapering schedule was flexibly customized. After drug withdrawal, constant medical assistance was maintained up to the end of the elimination process and until the patient’s post-elimination condition was normalized.

Therefore, the procedure was divided into 4 stages.

Stage I (substitution): apart from the commonly adopted reasons for a conversion to diazepam, the purpose specific for this study was a choice of a uniform drug for laboratory and statistical analyses. Preferably, the substitution was completed during the first 24 h. The initial (loading) dose of the substitute was roughly estimated using equivalency tables [28], but it could be completed by a titration procedure up to the patient’s (reported and observed) satiation state. That state, adopted as a baseline, was quantified by both the Clinical Institute Withdrawal Assessment Scale—Benzodiazepines (CIWA-B) score [30] and the corresponding satiating serum-BZD concentration according to the assay typically available (SBENZ immunoassay/COBAS integra 400 plus analyzer, Roche Diagnostics, LoD 3 ng/mL, precision 5.5% CV, accuracy 100% for negative samples, 100% for GC/MS-positive samples, 8/74 samples with SBENZ-positive/GC/MS-negative result) [31].

Stage II (counteracted accumulation): having established the satiating dose, to minimize further (unneeded) ascent of concentration due to superimposing consecutive doses, the dose was reduced in daily steps roughly estimated from laboratory feedback, starting with a 25% step. The serum-BZD measurements were performed daily, prior to the morning dose, until accumulation stopped.

Stage III (elimination): with initiation of the effective elimination process, especially after descent below the concentration baseline, the tapering rate became slower and flexibly driven by the current intensity of withdrawal symptoms (slowed or even suspended at symptom crises until adaptation improved). The patients were examined daily, while the CIWA-B questionnaire they completed at any reported change. Serum checks were performed every 3–7 days to track the elimination course. After drug withdrawal, the concentration and CIWA-B-score measurements were continued. The serum-BZD decline below the trackable level (< 3 ng/ml) was tentatively adopted as the end of the stage.

Stage IV (post-elimination observation): an inpatient assistance mode was continued until satisfactory adaptation to the abstinence, referring to the patient’s CIWA-B baseline.

During the treatment, the withdrawal symptoms were alleviated by adjunct medication adjusted to currently dominating ailments. Drugs that might significantly influence the elimination process were avoided in this sample.

### Data elaboration and statistical analysis

Regardless of the flexible character of the procedure, the course of the detoxification could be described by some essential elements, such as the initial (loading) diazepam dose and the resulting baseline concentration, the day and the level of maximal serum-BZD accumulation (D_ACC_, C_ACC_) indicating the start of the effective elimination process, the withdrawal day D_W_ (next day after the last administered dose), the elimination day D_E_ (trackable elimination completed), and the days of withdrawal crises.

The days of crises were indicated by extrema within a sequence of the CIWA-B scores accompanying the patient’s report of a perceptible worsening. For analyses, the days of the maximal and the last crisis (D_MAX_, D_LAST_) were adopted. The exact values of CIWA-B scores, as composed mainly (17/20 items) of patients’ highly subjective symptom-intensity ratings, were not comparable between the patients and not included in the group analyses.

For calculations, unless otherwise stated, days of relevant events should be understood as the day when an event occurred, counted from the beginning of the procedure. To better understand the time relationships between clinical events and the detoxification course, some data, if relevant, could be additionally related to D_ACC_ as a day starting an effective elimination process. For practical illustration of the results, some events also referred to the day of drug withdrawal.

The results were compared between male and female patients and between those who received elimination-modifying medications and those who did not. The treatment-related data referred to the extrema of withdrawal symptoms, as well as to the patient’s sex, age and duration of addiction (years of chronic BZD intake).

Due to the asymmetrical distribution of some data, apart from the average and the standard deviation (SD), the median, maximal and minimal values are presented. For analyses, nonparametric tests were applied: the Mann–Whitney test for intergroup comparisons and the Spearman test for correlations between relevant patient- and detox-related data. The statistical tests were performed using Statistica 13.3 [32]

## Results

Detoxification was completed by 321 patients, representing 91.7% of the initial sample. The enrolled group and concurrent medication characteristics – see Suplements A-D.

The considerable bulk of patients entered the study receiving carbamazepine (108, 33.6%) or valproates (127, 39.6%), which (respectively) accelerated or slowed BZD metabolism, making up subgroups for comparisons of the elimination data (Table 1).

The satiation state (stage I) was achieved at an average diazepam dose of 27.4 mg (SD 25.4, or median 22, range 2–220). Stage II actions resulted in the cessation of serum-BZD accumulation on approximately the 5th day (5.0 SD 3.9 or median 5, 1–12) of the procedure (D_ACC_) at the median peak C_ACC_ levels of 554, 52–4763 ng/ml (525, 52–4763 ng/ml in women and 608, 75–4760 ng/ml in men, non-significant difference, ns). To achieve this, the dose had to be reduced in daily steps varying under laboratory feedback between 1/6 and 1/3 of the previous dose. In detail, issues related to accumulation counteraction will be discussed in a separate article.

The elimination stage (III), starting with markedly reduced doses, lasted an average of 29.1 days (SD 14.3, median time 26, 5–96), including 22.3 (SD 13.7, median time 19, 2–95) days after drug withdrawal, with no differences between males and females. On average, trackable elimination was completed on day 34 (34.5 SD 14.8, median 30, 6–105) day of the study. The comparison with respect to concomitant use of elimination modifiers suggested the longest elimination in the valproate group (Table 1), but that result reached significance only when compared with the carbamazepine group, and in the t-test only. Moreover, the patients who eliminated the longest were outside the valproate group.

The withdrawal syndrome (within stages III and IV) culminated several times, with varying combinations of symptoms. Their maximal intensity occurred on average on day 20 (19.7 SD 13.3, median 17, 5–79), while the last peak (which was also the maximal one in 145 (45.2%) cases) on the 30th (30.2 SD 16.7, median 28, 5–99) day of the procedure, with no significant differences between the sexes. When related to drug withdrawal, the last crisis occurred an average of 18 days (17.9 SD 15.7, or median 15, -11–84) afterwards.

The days of crisis (Table 2) correlated with age, and the last one correlated with the duration of addiction. Above all, however, the crisis days, particularly the last one, were highly correlated with the day of the concluded elimination, whether counted from the beginning of the procedure (as “elimination day”, D_E_) or from the start of the elimination stage (“elimination duration” D_E_ – D_ACC_). A similar dual time reference applied for the days of crisis (Table 3) revealed that the correlation was the strongest when all correlating days were counted directly. The correlation was confirmed in the elimination-modifier subgroups, reaching the highest significance in the valproate group (Table 3). The other crisis correlates in Table 2 are probably secondary to those shown in Table 4.

**Table 1:** Modification of the elimination duration in the carbamazepine (CBZ) and valproate (VAL) groups compared directly (last column) and to the elimination data for patients (NONE) who were not taking either drug. The average (AVG) and median (MED) time (days) elapsed from the start of the procedure (D_E_), from the start of the elimination stage (D_E_ – D_ACC_), or from the BZD withdrawal (D_E_ – D_W_) were compared using parametric and non-parametric tests, respectively. Abbreviation ns means non-significant difference

Elimination data		CBZ tests vs. NONE	NONE	VAL tests vs. NONE	CBZ vs. VAL
Elimination day (D_E_)	AVG (STD) t, p MED (MIN–MAX) Z, p	32.76 (12.61) ns 30 (15–105) ns	34.05 (16.54) - 29 (6–93) -	36.61 (15.21) ns 33 (13–79) ns	2.10, 0.037 ns
Elimination duration (D_E_ – D_ACC_)	AVG (STD) t, p MED (MIN–MAX) Z, p	27.45 (12.02) ns 25 (11–96) ns	28.00 (15.85) - 23 (5–85) -	31.16 (14.84) ns 28 (6–71) ns (0.053)	2.09, 0.038 ns
Elimination after the drug withdrawal (D_E_ – D_W_)	AVG (STD) t, p MED (MIN–MAX) Z, p	20.49 (12.16) ns 18 (4–95) ns	21.31 (14.42) - 18 (2–72) -	24.36 (14.19) ns 21 (4–67) ns	2.23, 0.027 ns﻿

**Table 2:** Variables correlating with the days of the procedure (D_MAX_, D_LAST_), when the maximal (MAX) and the last (LAST) withdrawal crises occurred. Spearman’s rho value and significance are presented. The correlations secondary (similar but weaker) to those listed in Table 4 are in italics

SPEARMAN’S CORRELATION ρ, p	ELIMINATION FINAL DAY (D_E_)	ELIMINATION DURATION (DACC – DE)	ELIMINATION START DAY (D_ACC_)	WITHDRAWAL DAY (D_W_)	AGE	YEARS OF ADDICTION
MAX CRISIS (D_MAX_)	+ 0.37 < 0.0005	+ 0.36 < 0.0005	ns	ns	+ *0.16* *0.004*	ns
LAST CRISIS (D_LAST_)	+ 0.61 < 0.0005	+ 0.58 < 0.0005	+ *0.20* < *0.0005*	+ *0.23* < *0.0005*	+ 0.27 <0.0005﻿	+ 0.25 < 0.0005

**Table 3:** The overall correlation between the peak days of the withdrawal crises (the maximal and the last one D_MAX_, D_LAST_, respectively) and the elimination-end data, verified in subgroups taking concomitant elimination-modifying medication (CBZ, VAL or NONE) and analysed using the dual time-reference: days counted either from the start of the procedure (D_E_, D_MAX_, D_LAST_) or from the start of elimination stage (D_E_ – D_ACC_, D_MAX_ – D_ACC_, D_LAST_ – D_ACC_). Abbreviations the same as in Table 1 The correlation was the most apparent when all the days were counted directly (highlighted in bold) and not related to other (D_ACC_) events. The correlation was the highest in the VAL group.

Spearman’s correlation	Maximal crisis day		Last crisis day	
	D_MAX_	D_MAX_ – D_ACC_	D_LAST_	D_LAST_ – D_ACC_
CBZ				
Elimination day (D_E_)	**ρ**_**3.54**_** = 0.32** **0.006**	ρ_2.34_ = 0.22 0.02	**ρ**_**7.10**_** = 0.57** ** < 0.0000005**	ρ_6.10_ = 0.51 < 0.0000005
Elimination duration (D_E_ ﻿– D_ACC_)	ρ_3.53_ = 0.32 0.006	ρ_3.48_ = 0.32 0.0007	ρ_6.42_ = 0.53 < 0.0000005	ρ_6.88_ = 0.56 < 0.0000005
NONE				
Elimination day (D_E_)	**ρ**_**3.28**_** = 0.34** **0.002**	ρ_1.93_ = 0.21 0.057 (ns)	**ρ**_**6.73**_** = 0.60** ** < 0.0000005**	ρ_5.55_ = 0.53 < 0.0000005
Elimination duration (D_E_ – D_ACC_)	ρ_3.07_ = 0.32 0.003	ρ_2.74_ = 0.29 0.007	ρ_8.44_ = 0.58 < 0.0000005	ρ_6.51_ = 0.58 < 0.0000005
VAL				
Elimination day (D_E_)	**ρ**_**5.48**_** = 0.44** ** < 0.0000005**	ρ_4.70_ = 0.39 0.000007	**ρ**_**10.87**_** = 0.69** ** < 0.0000005**	ρ_9.68_ = 0.65 < 0.0000005
Elimination duration (D_E_ – D_ACC_)	ρ_5.29_ = 0.43 < 0.0000005	ρ_5.46_ = 0.44 < 0.0000005	ρ_9.65_ = 0.65 < 0.0000005	ρ_9.89_ = 0.66 < 0.0000005

Table 4, in turn, concerns the two main correlates of the withdrawal crises, i.e., the elimination day and the elimination duration. As expected, data related to the final elimination correlated with other elimination-related data, such as elimination start data (D_ACC_ and C_ACC_)*,* the duration of sustained drug administration and the day of drug withdrawal D_W_. The elimination day and duration also correlated with patient age and duration of addiction.

Table 5 presents the remaining correlations among the variables in the study, commented on in the discussion below.

**Table 4:** Variables correlating with the elimination final day and duration of effective elimination as the two main correlates of the crisis days (c.f. Table 2)

SPEARMAN’S CORRELATIONS (ρ, p) BETWEEN:	ELIMINATION FINAL DAY (D_E_)	ELIMINATION DURATION (D_E_ – D_ACC_)
AGE	+ 0.28 < 0.0005	+ 0.30 v < 0.0005
YEARS OF ADDICTION	+ 0.23 < 0.0005	+ 0.24 < 0.0005
ELIMINATION START LEVEL (C_ACC_)	+ 0.18 < 0.002	+ 0.20 < 0.0005
ELIMINATION START DAY (D_ACC_)	+ 0.30 < 0.0005	ns
WITHDRAWAL DAY (D_W_)	+ 0.35 < 0.0005	+ 0.22 < 0.0005
EFFECTIVE TAPERING TIME: FROM ELIMINATION START TO THE WITHDRAWAL (D_W_ – D_ACC_)	+ 0.19 < 0.0005	+ 0.23 < 0.0005

**Table 5:** Remaining correlations among the variables in the study. Spearman’s ρ and p are presented

	YEARS OF ADDICTION	MAXIMAL CONCENTRATION LEVEL (CACC)	WITHDRAWAL DAY	EFFECTIVE TAPERING TIME
AGE	+ 0.34, < 0.0005	-0.19, < 0.0005	-0.12, 0.029	-0.11, 0.038
MAXIMAL CONCENTRATION LEVEL (C_ACC_)		xxxxxxxxxxxxxxx	+ 0.34, < 0.0005	+ 0.41, < 0.0005
MAXIMAL CONCENTRATION DAY (D_ACC_)			+ 0.42, < 0.0005	-0.14, 0.012
EFFECTIVE TAPERING TIME			+ 0.79, < 0.0005	xxxxxxxxxxxxxxx

## Discussion

This study supports the hypothesis [25] that one of the possible reasons for early relapses may be customary premature conclusions of detoxification, followed by delayed drug elimination and the related withdrawal crisis.

**Fig. 1:**
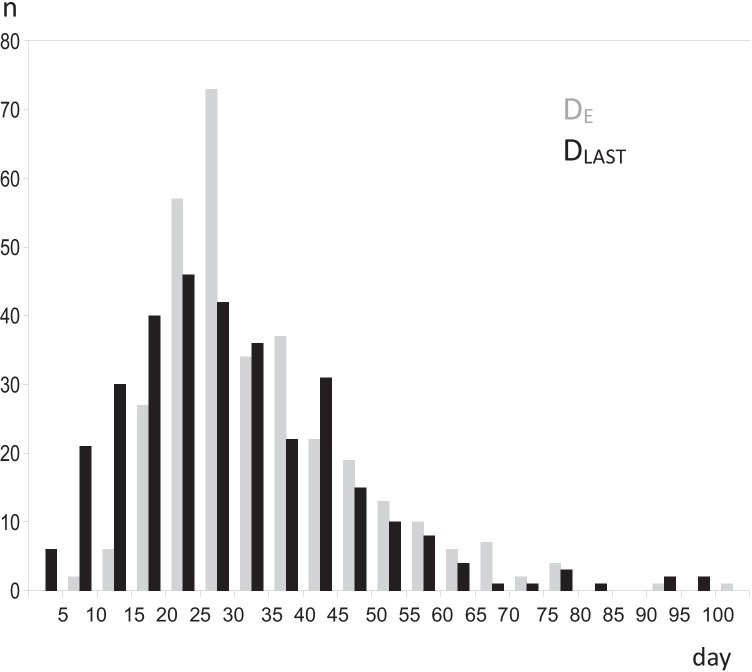
Superimposed histograms of time to the final day of elimination (D_E_, gray bars) and to the peak day of the last withdrawal crisis (D_LAST_, black bars), counted from the start of the procedure. The vertical axis label (n) indicates the number of patients who had their D_E_ (or D_LAST_, respectively) occurring within a given 5-day time frame (the horizontal axis)

The timing of the crises, especially of the last one, correlated with the final elimination stage (Table 2) regardless of the dual time reference adopted. However, the correlation was most evident when the days were not related to other events (Fig. 1, Table 3).

Completion of elimination, in turn, has been shown to be considerably delayed relative to drug withdrawal. This should not come as a surprise given the diazepam half-life of 36–200 h, including active metabolites [15]. It needs to be emphasized that in this study, despite the action against unneeded accumulation, the BZDs persisted in blood an average of 2–3 weeks after drug withdrawal but much longer in some cases (3 months and more)*,* especially in older patients (Table 4). In traditional BZD-detox approaches, using diazepam with no anti-accumulation corrections provided, the day of elimination must be delayed even more, as it is dependent on both the initial level (highly accumulated) and the initial day (delayed) of the effective elimination process.

The main result, which is the significant time relationship between the last crisis and the final elimination process, is consistent with observations suggesting a low-concentration trigger for decisive discontinuation symptoms [11, 13, 14]. However, without serum concentration measurements, it is difficult to estimate which culmination of symptoms could be the last one. Usually, following several consecutive crises when patients overcome successive re-adaptation thresholds, a patient’s good mood after the final drug withdrawal encourages the detox conclusion. One more crisis resulting from delayed concentration descent may then occur after cessation of medical assistance. This, in turn, may evoke a relapse.

The study pointed at some factors that may influence elimination delay and the related delay of the last crisis. These factors may be patient- or detox-related.

The patients’ age (Table 4) is an expected factor, as with increasing age, metabolism slows down and elimination lasts longer. That age-related slowing outweighed the fact that older patients needed less drug to achieve satiation (Table 5) and started the effective elimination from lower concentration levels.

The duration of addiction is linked with the crises in a less obvious way. It correlates with crises slightly more directly than with elimination data. One might say that longer addiction fixes drug-related adaptation, so re-adaptation is harder, resulting in protracted withdrawal symptoms. In this case, the parallel correlation with elimination data could be explained, for example, as longer elimination of more tissue-BZD deposits accumulated with time of addiction [33]. However, the correlation coefficients between the duration of addiction and both the last crisis (Table 2) and the elimination data (Table 4) are broadly similar, while the correlation with patient age is higher (Table 5). Obviously, longer addiction requires a longer life. Age, in turn, correlates with longer elimination, which indirectly links the duration of addiction with pharmacokinetics (PK). The proportions of various mechanisms underlying this complex relationship require further study. However, it is crucial to differentiate between protracted crises due to re-adaptation difficulties and crises in the course of “normal” re-adaptation, which is simply delayed due to PK phenomena.

Apart from patient-related factors, specifics of detoxification play an important role in the timing of delayed crises. The elimination day and elimination duration (the main correlates of the crises) themselves expectedly correlate with other elements specific to the elimination course (Tab 3). The later the effective elimination starts and the higher the initial level is, and the later the tapered drug is withdrawn, the later the elimination process comes to an end. Some of these relationships are mirrored as respective but weaker correlations with days of crisis (Table 2). Hence, it is the end of the elimination phase and not the end of drug administration, for example, that is most directly related to the delay of the last withdrawal crisis.

Consequently, rigid tapering schedules leading to unprevented initial accumulation of a long-acting BZD delay the end of elimination and decisive withdrawal crisis. Furthermore, auxiliary medications, such as valproates, tend to prolong the BZD elimination course (Table 1). The time correlation between the end of elimination and the last crisis, occurring in each subgroup, is the most significant in the valproate group (Table 3), which makes the correlation all the more visible the longer the elimination process is extended.

It might be argued, however, that if the final withdrawal crisis is indeed closely related to the end of the elimination process, delayed elimination could be an advantage rather than a problem. Delayed crises, even if unpredicted, should be easier to pass due to more time for re-adaptation. However, this rule works only if it is the actual concentration-decline process that is extended (stage III here), occurring below the satiating (and not an over-accumulated) concentration level. As has been discussed elsewhere, in typical trials, this may not be the case [25].

The modified procedure, introduced here to prevent excessive accumulation of diazepam (stage II), actually boiled down to empirical determination of an approximate maintenance dose. As a result of several consecutive cuts by 1/6 to 1/3, this dose was usually reduced much more than the typical 10–25% used as the first tapering step [34] While the detailed analysis considering the accumulation stage will be provided in a separate paper, this result is obvious given the diazepam half-life. Thus, proper detoxification (effective elimination) started only when the patients were already placed at an advanced stage of dose reduction. In most approaches, however, when the initial tapering steps are cautiously small (no lab control) or/and preceded by “stabilization” (a common practice [24] when the loading dose is maintained for several days prior to the start of the tapering procedure), for some individually different times, the serum-BZD will still be increasing. Eventually, after achieving a non-optimal plateau, which transiently drives adaptation in the direction opposite to the treatment goal, effective elimination can start, but with a marked delay and from an excessive level. Thus, a later crisis may not mean a weaker crisis if the delayed elimination end results not from extension of the elimination process but from the delayed start only [25].

Consequently, a significant tapering deceleration to smoothen the re-adaptation process, although not applied in this study as it was focused on elimination issues, may be definitely advantageous, but only when it runs below the satiating concentration level, when re-adaptation mechanisms are stimulated.

Acknowledging that PK variability require individualized detox procedure, paves the way for further research on the underlying phenomena, including genetic (single-nucleotide) polymorphism, and their possible relevance for clinical practice.

### Limitations

The innovative flexible protocol of the study may be also perceived as controversial. Furthermore, an adjunct medication was allowed and differed between the patients. The seminaturalistic study, carried out within the reality of a typical detoxification ward, imposed medical and ethical obligations to optimize the treatment. However, these apparent limitations are actually advantages of the study.

Considering individual differences in both metabolism and re-adaptation rates, detoxification had to be customized and not scheduled. Moreover, the flexible procedure prevents systematic errors possible with a uniform schedule. The time correlation between the end of the trackable elimination process and the last observed withdrawal crisis, evident regardless of varying other settings, is not an artefact.

Furthermore, the results are relevant and replicable in real treatment settings. The replicability premise is recognition that it is the serum-BZD concentration and not the current dose that reflects the real progress of the detoxification process. Serum-BZD measurements both establish the nodal points of the detox procedure and enable its customization. As it is the resulting concentration that is measured (already including individual factors or effects induced by auxiliary medication), any adjustments based on laboratory feedback automatically include corrections for any (recognized or not) elimination modifiers. The valproate-dependent prolongation of elimination was most contributed by the post-withdrawal phase, when corrective changes in BZD tapering rate were no longer possible (Table 1). Most importantly, laboratory data indicate the patient’s entrance into the crucial, low-concentration phase.

The application of an immunoassay for serum-BZD level measurement may be both an advantage and a limitation of the study. As a standard laboratory test, it adjusts the study to a daily routine (including costs) of a detoxification ward. However, it is a limitation due to the lower precision of quantitative measurements compared with high-performance liquid chromatography (HPLC).

First, the immunoassay measures “benzodiazepines” in bulk, while any sample may contain diazepam and the metabolites nordiazepam, temazepam and oxazepam in evolving mutual proportions. These BZDs reveal good cross-reactivities with the test compounds, but transitions between the molecules result in serum-BZD fluctuations due to molecular mass changes. The maximal error in the extreme but practically unlikely situation of transition between samples containing 100% of the lightest (270.7 Da) and 100% of the heaviest (300.7 Da) BZD would be 10%, so in practice, it is lower. When the goal is to stop overaccumulation, an error of several percent is acceptable.

Second, tracing the final elimination process may be inaccurate due to the lower sensitivity of the assay at low concentrations. However, the possibility of false-negative results (despite the nominally completed elimination some active metabolites still present) would work even more in favour of the prolonged elimination claimed in this study. Thus, it would also strengthen the recommendation of extended, post-elimination medical assistance (stage IV here). The inactive metabolites (glucuronides), also detected in the test used, may increase the reading and delay the D_E_ indication. Their presence, however, does not destroy the observed correlation, as it can only shift the exact relationship in time (the data suggest that the crisis usually precedes the D_E_ by several days). False-positive artefacts (due to other substances, while BZDs are actually completely eliminated) appear less likely because the laboratory data (residual presence in blood) closely coincided with the clinical phenomena (delayed crises) regardless of auxiliary medication.

In theory, complete elimination will never occur, as the concentration approaches zero asymptotically. However, it may be hypothesized that the final re-adaptation may be established and crises stop recurring only when the concentration ceases to show a perceptible decrease, below the last measurable level. The test accuracy, although limited, is sufficient to estimate this moment, while clinical criteria alone are not. As some patients experienced several peaks of withdrawal symptoms, while others only experienced them in the advanced (low concentration) stage of the elimination process, neither any apparently past crisis nor the low severity of symptoms excludes a later breakthrough.

### Concluding remarks

The postulate resulting from the study to extend medical assistance beyond the completed BZD elimination does not require extended detoxification but only its optimization. Laboratory feedback, minimizing a high-concentration detox phase, expeditiously brings the tapering procedure to the stage at which deceleration becomes purposeful. Utilizing the time initially saved to extend the support throughout the entire low-concentration phase may prevent relapses due to unassisted delayed withdrawal crises.

For several years when this approach was developed, among several hundred patients total (the number higher than systematized in the present study), only 3 patients revealed evident protracted symptoms (extended far beyond the elimination end). These eventually ceased within 2 years of further care in ambulatory settings. This observation, added to the results of the study, leads to the conclusion that among those of early relapses that result from recurring withdrawal crises, the vast majority may be due not to re-adaptation problems but to prolonged elimination processes. Such delayed crises, as related to pharmacokinetic issues, are predictable and manageable. Concern for the hitherto underestimated elimination delay may definitely improve the treatment prognosis.

## Supplementary information

Below is the link to the electronic supplementary material.

Supplementary file1 (DOCX 6 KB)

Supplementary file2 (DOCX 6 KB)

## Data Availability

The datasets generated or analyzed during the current study are not available publicly due to local data protection regulations but available from the author on reasonable request.
