# Supplementary material for: Delayed crises following benzodiazepine withdrawal: deficient adaptive mechanisms or simple pharmacokinetics? Detoxification assisted by serum-benzodiazepine elimination tracking
Source: Eur J Clin Pharmacol. 2021 Sep 13;78(1):101–10. doi: 10.1007/s00228-021-03205-x (PMC8724079; doi:10.1007/s00228-021-03205-x)
Supplement: Supplementary file 1 — Supplementary file1 (DOCX 6 KB) [file 228_2021_3205_MOESM1_ESM.docx]

Supplement A

Characteristics of the group entering the study. In some patients more than one co-morbid disorders could have been diagnosed.

Abbreviations: SD - standard deviation, ns – non-significant difference. d. - disorder

|  | Women (W) | Men (M) | Total | Differences |
| --- | --- | --- | --- | --- |
| Participants (%)  Drop-outs (%) | 186 (53)  14 (7.5) | 164 (47)  15 (9.1) | 350 (100)  29 (8.3) |  |
| Age:  average (SD)  median (min-max)  Patients > 65 years | 50.5 (14.9)  52 (20-87)  21 | 47.4 (14.8)  49 (20-86)  18 | 49.1 (13.8)  49 (20-87)  39 | ns  ns |
| Years of BZD addiction: average (SD)  median (min-max) | 11.5 (9.0)  10 (0.6-50) | 9.8 (8.3)  8 (0.2-40) | 10.6 (8.5)  9 (0.2-50) | ns  ns |
| Co-morbid disorders (cases):  alcohol dependence  anxiety disorders:  - panic anxiety d.  - generalized anxiety d.  - other/mixed anxiety d.  mood disorders  - bipolar disorder  - major depressive d.  - other depressive disorders  personality/behaviour d.  primary insomnia | 42  18  24  41  28  35  6  23  7 | 80  23  20  15  30  18  18  43  10 | 122  41  44  56  58  53  24  76  17 |  |
