# Supplementary material for: Delayed crises following benzodiazepine withdrawal: deficient adaptive mechanisms or simple pharmacokinetics? Detoxification assisted by serum-benzodiazepine elimination tracking
Source: Eur J Clin Pharmacol. 2021 Sep 13;78(1):101–10. doi: 10.1007/s00228-021-03205-x (PMC8724079; doi:10.1007/s00228-021-03205-x)
Supplement: Supplementary file 2 — Supplementary file2 (DOCX 6 KB) [file 228_2021_3205_MOESM2_ESM.docx]

Supplement B

The most frequent psychiatric medications applied in the study participants in the period preceding detoxification (accepted and continued throughout the study). In particular, the anticonvulsive agents, formerly introduced, could not be withdrawn at the detox onset.

| Medication | Cases | Condition | Application |
| --- | --- | --- | --- |
| valproates | 127 | bipolar mood disorder  other mood disorders  benzodiazepine dependence | mood stabilizer (normothymic)  adjunct medication  anticonvulsant (securing against witdrawal seizures) |
| carbamazepine | 108 | mood disorders  personality/behavioral d.  benzodiazepine dependence | mood stabilizer, adjunct med.  adjunct medication  anticonvulsant |
| venlafaxine | 24 | anxiety disorders  mood disorders | anxiety treatment  antidepressant |
| sertraline | 10 | anxiety disorders  mood disorders | anxiety treatment  antidepressant |
| es- or citalopram | 11 | anxiety disorders  mood disorders | anxiety treatment  antidepressant |
| paroxetine | 4 | anxiety disorders | anxiety treatment |
| tianeptine | 10 | mood disorders | antidepressant |
| mianserin | 25 | mood disorders | antidepressant |
| agomelatine | 22 | mood disorders | antidepressant |
| lamotrigine | 11 | bipolar mood disorder | mood stabilizer |
| olanzapine | 12 | bipolar mood disorder  depressive mood disorder | anti-manic agent  adjunct and sleep-promoting drug |
| quetiapine | 34 | bipolar mood disorder  depressive mood disorder | anti-manic agent  adjunct medication |
| aripiprazol | 15 | bipolar mood disorder | anti-manic drug |

Supplement C.

The most frequent permanent medication of somatic illnesses in the study participants. as accepted and continued throughout the study.

| Medication | Condition |
| --- | --- |
| enalapril, ramipril  telmisartan  amlodipine  bisoprolol | arterial hypertension |
| rova- or atorvastatine | hypercholesterolemia |
| levothyroxine | hypothyroidism |
| doxazosine | prostatic hypertrophy  arterial hypertension |

Supplement D.

The most frequent adjunct medication temporarily applied to alleviate BZD-withdrawal symptoms. The drugs were used on demand or temporarily applied on a regular basis.

| Medication | Symptoms |
| --- | --- |
| captopril  atenolol  propranolol  magnesium  ibuprofen | elevated arterial pressure  tachycardia, elevated arterial pressure  tachycardia, tremor  tachycardia, tremor, increased muscle tension  headache, muscle pain |
| pantoprazol  loperamide | gastrointestinal problems |
| hydroxyzine  promethazine  tiaprid  quetiapine | agitation, anxiety, sleep disturbances |
| trazodon  mianserin  mirtazapine  promazin | sleep disturbances |
| carbamazepine | neuralgia, paresthesia |
